# Human umbilical cord-derived mesenchymal stem cells attenuate hepatic stellate cells activation and liver fibrosis

**DOI:** 10.1007/s11033-024-09664-6

**Published:** 2024-06-14

**Authors:** Xiaoyu Shi, Kun Zhang, Qi Qi, Wangyi Zhou, Fengshi Yu, Yu Zhang

**Affiliations:** 1 State Industrial Base for Stem Cell Engineering Products, Tianjin, 300384 China; 2 Tianjin Key Laboratory for Stem Cell and Regenerative Medicine, Tianjin, China; 3 VCANBIO Cell & Gene Engineering Corp., Ltd, Tianjin, China; 4 Tianjin Key Laboratory for Blood Cell Therapy Technology, Tianjin, China; 5 Haihe Laboratory of Cell Ecosystem, Tianjin, China

**Keywords:** UC-MSC, MSC conditional medium, Hepatic stellate cell activation, CCL_4_-induced liver fibrosis, Immunomodulation

## Abstract

**Background:**

Liver cirrhosis, a prevalent chronic liver disease, is characterized by liver fibrosis as its central pathological process. Recent advancements highlight the clinical efficacy of umbilical cord mesenchymal stem cell (UC-MSC) therapy in the treatment of liver cirrhosis.

**Methods and results:**

We investigated the pharmacodynamic effects of UC-MSCs and MSC conditional medium (MSC-CM) in vivo, utilizing a carbon tetrachloride (CCl_4_)-induced fibrotic rat model. Concurrently, we assessed the in vitro impact of MSCs and MSC-CM on various cellular process of hepatic stellate cells (HSCs), including proliferation, apoptosis, activation, immunomodulatory capabilities, and inflammatory factor secretion. Our results indicate that both MSCs and MSC-CM significantly ameliorate the pathological extent of fibrosis in animal tissues, reducing the collagen content, serum biochemical indices and fibrosis biomarkers. In vitro, MSC-CM significantly inhibited the activation of the HSC line LX-2. Notably, MSC-CM modulated the expression of type I procollagen and TGFβ-1 while increasing MMP1 expression. This modulation restored the MMP1/TIMP1 ratio imbalance and extracellular matrix deposition in TGFβ-1 induced fibrosis. Both MSCs and MSC-CM not only induced apoptosis in HSCs but also suppressed proliferation and inflammatory cytokine release from activated HSCs. Furthermore, MSCs and MSC-CM exerted a suppressive effect on total lymphocyte activation.

**Conclusions:**

UC-MSCs and MSC-CM primarily modulate liver fibrosis severity by regulating HSC activation. This study provides both in vivo and in vitro pharmacodynamic evidence supporting the use of MSCs in liver fibrosis treatment.

**Supplementary Information:**

The online version contains supplementary material available at 10.1007/s11033-024-09664-6.

## Introduction

Liver fibrosis, a prevalent pathological outcome of various liver diseases, including viral hepatitis, alcoholic steatohepatitis, nonalcoholic steatohepatitis, drug misuse, and autoimmune hepatitis, arises as a wound-healing response to chronic liver injury [1]. Persistent inflammatory stimuli lead to an increase in liver extracellular matrix (ECM) production while concurrently hindering its degradation, ultimately resulting in excessive accumulation of ECM proteins. Failure to address this fibrosis effectively can lead to the progression of liver fibrosis to liver cirrhosis, which can give rise to numerous severe complications including liver failure or hepatocellular carcinoma [2]. Notably, hepatic stellate cell (HSC) activation plays a crucial role in the liver fibrosis pathogenesis [3]. HSCs, which reside in the peri- sinusoidal space between the hepatic cord and sinusoids, undergo activation and transdifferentiation into myofibroblasts upon exposure to sustained inflammatory insults. This process endows HSCs with contractile, pro-inflammatory, and fibrogenic properties, thus contributing significantly to the development and progression of liver fibrosis [4]. These activated HSCs lead to the amplification of ECM synthesis and suppression of its degradation, thereby causing excessive accumulation of ECM in the liver. Consequently, the prevention of HSC activation emerges as a promising therapeutic strategy for liver fibrosis treatment [5].

Mesenchymal stem cells (MSCs), pluripotent adult stem cells derived from the mesoderm, possess the remarkable ability to differentiate into multiple cell lineages. MSCs can be conveniently isolated from diverse sources, such as bone marrow (BM), adipose tissue (AT), and the umbilical cord (UC), exhibiting low immunogenicity [6]. The key therapeutic properties of MSCs lie in their robust self-renewal and engraftment capabilities. Increasing evidence elucidates MSCs’ potential in secreting multiple bioactive molecules with distinct physiological functions, making them beneficial for liver disease management. These bioactive molecules encompass cytokines, receptors, transporters, and binding proteins, all playing pivotal roles in processes such as proliferation, differentiation, immunomodulation and apoptosis [7, 8]. MSCs exhibit remarkable immunomodulatory and anti-inflammatory capacities, enabling them to suppress T and B lymphocyte proliferation, promote cytokine release and attenuate inflammatory cytokine synthesis. Furthermore, through chemotaxis, MSCs can be precisely directed to liver injury sites, where they potentiate hepatocyte paracrine activity via critical factors like hepatocyte growth factor (HGF), insulin-like growth factor (IGF) and interleukin 10 (IL-10), thereby bolstering the repair and regeneration of damaged liver tissue [9, 10]. Notably, numerous preclinical studies have proved the potential of MSCs from different tissues, such as adult-derived human liver MSCs (ADHLSCs), bone marrow-derived MSCs (BMSCs) to safely ameliorate liver cirrhosis and enhance liver functionality [11–13]. UC-MSCs have also exhibited significant efficacy in ameliorating liver functionality and clinical manifestations among patients with cirrhosis, by optimizing key parameters like serum bilirubin and transaminases levels, Child-Pugh scores, as well as enhancing serum albumin concentrations. Additionally, these cells have been shown to diminish complications associated with ascites and portal hypertension [14]. Currently, MSCs are administered either systemically or locally, with intravenous infusion being the clinical preferred approach. Since the average diameter ranging from 14 μm to 20 μm in a suspension state, MSCs exceed the size of pulmonary capillaries, resulting in their non-specific pattern. Over time, the retention of MSCs in the lungs gradually decreases, while a proportion of MSCs migrate to the liver and spleen [15]. The majority of infused cells are eliminated by the circulatory system, with only a minor fraction successfully homing to the target organs and exerting therapeutic functions. In light of this, it is imperative to investigate the therapeutic efficacy of non-viable cell preparations, including extracellular vesicles (EVs) derived from MSCs or MSC-conditioned medium (MSC-CM). These preparations could potentially offer a promising alternative to direct MSC therapy for various ailments treatment. In summation, it becomes imperative to develop innovative UC-MSC therapeutic strategies to counteract HSC activation, particularly in fibrotic hepatic conditions such as liver fibrosis and cirrhosis. The precise mechanisms underlying how MSCs regulate HSC activation remain enigmatic and require to be elucidated.

In our research, we examined the effects of UC-MSC-CM, enriched with soluble factors from MSCs, on CCL_4_-induced liver fibrosis in rats. Our results focused on histopathological alterations in the liver, serological markers indicative hepatic fibrosis, serum biochemical indices and protein expression of fibrosis biomarkers. These parameters were critical for understanding the pathogenesis of liver fibrosis. Our results demonstrated that the administration of MSC-CM to rats with liver fibrosis promoted recovery comparable to that achieved with live MSC infusion. Mechanistically, MSC-CM modulated TGFβ-1 activated HSCs by suppressing pro-fibrogenic reactions. This was evident by a decrease in collagen type I alpha 1 chain (COL1A1) and TGFβ-1 expression and an increase in matrix metalloproteinase 1 (MMP1) expression at both mRNA and protein levels. Treatments with MSC-CM, as well as co-culture with UC-MSCs, effectively inhibited HSC proliferation while promoting their apoptosis. Furthermore, MSC-CM effectively mitigated chronic liver fibrosis by suppressing inflammatory mediators and curbing the proliferative potential of total lymphocytes.

## Materials and methods

### Isolation and culture of UC-MSCs

The study was granted approval by the Clinical Research Ethics Committee of Renmin Hospital of Wuhan University, with the approval number WDRY2019-Q026 granted on date 20/12/2019. UCs were generously donated by consenting mothers for research purposes and were harvested post-disinfection. Written informed consent was obtained from mothers to ensure ethical compliance. These umbilical cords were meticulously sectioned into segments measuring 1–2 cm. After a meticulous cleaning process, the segments were diced into tissue blocks of 1–2 mm³. These tissue blocks were then cultured in Dulbecco’s modified eagle medium (DMEM)/F12 medium with 10% fetal bovine serum (FBS, purchased from Biological Industries, ART NO.04-001-1ACS), and maintained at 37℃in a 5% CO_2_ humidified incubator. Once the MSCs started migrating, the medium was refreshed every 2–3 days to ensure continuous cell growth. Once the cells reached 80-90% confluence, they were subcultured using a 0.25% trypsin-ethylenediaminetetraacetic acid (EDTA) solution. Following this, a comprehensive analysis was conducted to assess the phenotype and multipotent attributes of the MSCs.

### Preparation of MSC-CM

UC-MSCs were seeded at a density of 2 × 10^4^/cm^2^ (totally 1.5 × 10^6^ cells)in T75 flasks containing 15 mL complete culture medium. Roughly 6–8 h after seeding, the cells were gently rinsed twice with phosphate-buffered saline (PBS), and the medium was subsequently replaced with 15 mL DMEM/F12. The flasks were incubated for 48 h, then the supernatant was carefully collected. The supernatant was then centrifuged at a speed of 1000 rpm for 10 min to pellet the cells and debris. The remaining MSC cells in the flasks were also calculated by AOPI staining. The resultant MSC-CM was aliquoted into sterile tubes and stored at -80 °C for future use.

### Animal model, UC-MSCs and MSC-CM treatment

The Institutional Animal Care and Use Committee (IACUC) of Tianjin Tiancheng Drug Assessment Research Co., Ltd., granted approval for the animal experiment conducted in this study with approval no. 2,023,033,001, granted on 30/03/2023. All the animal experiments were conducted in accordance with the ARRIVE guidelines, ensuring transparency and ethical reporting of animal-based research. All methods utilized in this study were meticulously in strict accordance with pertinent guidelines and regulations to ensure ethical and accurate research practices.

The Wistar rats aged 29–35 days, with a weight range of 160–180 g, were purchased from Beijing Vital River Laboratory Animal Technology Co., Ltd (China). To induce liver fibrosis, the male Wistar rats received subcutaneous injections of a 40% carbon tetrachloride (CCl_4_) solution in olive oil. Initially, the dose was set at 5 mL/kg, and subsequently reduced to 3 mL/kg for bi-weekly administrations over a period of 11 weeks. Meanwhile, throughout the modeling period, commencing from the third week until its conclusion, the rats were fed with a specialized diet high in fat and low in protein. This diet comprised 80% corn flour, 17.5% lard, 0.5% cholesterol and 2% sodium chloride. In contrast, the control group exclusively received olive oil injections alongside a normal feed. Based on the modeling phase outcomes, the rats were segregated into four distinct groups (with ten rats in each group): Control, CCl_4_, CCl_4_ + MSC-CM, and CCl_4_ + MSCs. The CCl_4_ + MSC-CM group received bi-weekly injections of 1.5 mL of MSC-CM per rat. Conversely, the CCl_4_ + MSCs group was treated with 2.5 × 10^6^ cells per rat weekly, spanning a three-week period following CCl_4_ administration. In contrast, both the control and CCl_4_ groups solely received equal volumes of PBS. Subsequently, following the administration of MSCs and MSC-CM, precisely on day 99 (D99) and two weeks later on day 113 (D113), the rats were anesthetized with 20% urethane and humanely euthanized by bleeding, in preparation for subsequent analyses.

### Liver histopathological assessment and fibrosis indices quantification

After the rats were humanely euthanized, extracted liver specimens were preserved in 10% neutral-buffered formalin. The paraffin-embedded tissue sections were then stained with hematoxylin and eosin (HE) and Masson’s trichrome for a thorough histopathological assessment of fibrosis. The collected serum was centrifuged at 3,000 rpm to assess the levels of various biochemical markers, including alanine aminotransferase (ALT), aspartate aminotransferase (AST), alkaline phosphatase (ALP), albumin (ALB), total bilirubin (TBIL), collagen type IV (COLIV), procollagen type III (PCIII), laminin (LN) and hyaluronic acid (HA).

Two weeks after the final administration, the liver tissues were excised and quantitatively evaluated for weight. The levels of TGFβ-1, α-SMA, COL I, and HYP protein in the liver tissue were determined using an enzyme-linked immunosorbent assay (ELISA).

### Culture of HSC and treatment with MSC-CM

The human HSC cell line LX-2 was obtained from iCell Company and cultured in DMEM medium supplemented with 10% FBS. To induce the activation of LX-2, they were seeded at a density of 2 × 10^4^/cm^2^ in DMEM/F12 medium, followed by treatment with TGFβ-1 at a concentration of 10 ng/mL for 12 h. For the MSC-CM treatment, the supernatant from activated LX-2 cells was carefully removed, followed by incubation of the cells in MSC-CM for additional 48 h. The experimental groups were designated as follows: (1) LX-2: quiescent LX-2 cells serving as the vehicle control; (2) LX-2 + TGFβ-1: activated LX-2 cells treated with TGFβ-1 as the positive control; (3) LX-2 + TGFβ-1 + MSC-CM: activated LX-2 cells treated with MSC-CM for 48 h, as the experimental group.

### Coculture of UC-MSCs with activated LX-2 cells

After activation, LX-2 cells were either cultured independently or co-cultured with UC-MSCs utilizing transwells designed for 24-well plates. In the lower chamber, the activated LX-2 cells were seeded at a density of 2 × 10^4^ cells per well, while the UC-MSCs were allocated in the upper chamber at densities of either 2 × 10^4^ or 2 × 10^5^ cells per well, depending on the desired target-to-effector ratio. The culture medium was DMEM/F12, and the LX-2 cells were harvested after co-cultured for 48 h.

### RNA extraction and quantitative assessment of fibrosis markers via real-time polymerase chain reaction (qRT-PCR)

Isolation of total RNA from the cells was performed using the TaKaRa MiniBEST Universal RNA Extraction Kit, followed by cDNA synthesis using the RevertAid First Strand cDNA Synthesis Kit (Thermo, K1622). qRT-PCR assays were conducted in triplicate with TB Premix EX Taq. The expression levels of the activation markers were determined based on the comparative threshold cycle value (2^−ΔΔCt^) method, with β-actin serving as the reference gene.

The primers sequences were as followed:

COL1A1-F: 5’-GCCAAGACGAAGACATCCCA-3’,

COL1A1-R: 5’-GGCAGTTCTTGGTCTCGTCA-3’;

TGFβ-1-F: 5’-CCTTCCCTCTGAACTCCTACAT-3’,

TGFβ-1-R: 5’-ATCGAAGTAGAGGACGGAGATG-3’;

MMP1-F: 5’-TGTTCTGGGGTGTGGTGTCT-3’,

MMP1-R: 5’-ACTGGGCCACTATTTCTCCG-3’;

β-actin-F: 5’-AGAGCTACGAGCTGCCTGAC-3’,

β-actin-R: 5’-ACAGCACTGTGTTGGCGTAC-3’.

### Immunofluorescence analysis

To assess the expression of collagen I in LX-2 cells across varying experimental groups, immunofluorescence staining was employed. Cells from different experimental groups were washed twice with PBS, fixed in 4% paraformaldehyde for 15 min, and then permeabilized with 0.5% Triton X-100 for an additional 15 min at room temperature. After triple PBS washes, the cells were blocked with 10% goat serum (Solarbio, SL038) in PBS for one hour. Then the cells were incubated overnight at 4 °C with the primary antibody Collagen I (1:500 dilution, Abcam, ab138492). On the subsequent day, after discarding the primary antibody and washing the cells thrice with PBS, they were incubated with the secondary antibody, goat anti-rabbit IgG H&L conjugated to Alexa Fluor 488 (Abcam, ab150077), in the dark for one hour. Following nuclear staining with 20% DAPI (CST, 8961 S, diluted in PBS) for 5 min, cellular images were captured using a fluorescence microscope.

### Enzyme-linked immunosorbent assay (ELISA)

The quantification of TGFβ-1, MMP1, IL-6, IL-8, and pre-collagen concentrations in cell culture supernatants was measured utilizing specific ELISA kits, namely: human pre-collagen (NBP2-76465, R&D), human TGFβ-1 (VAL127, Bio-techne), human MMP1 (ab215083, Abcam), human IL-6 (101,002, Novus), and human IL-8 (100,803, Novus). All kits were operated strictly in accordance with their respective protocols to ensure accurate measurements. Spectrophotometric measurements were performed to the optical density at 450 nm. The quantification of data was achieved in pg/mL by referencing the respective standard curves provided with each ELISA kit.

### Cell proliferation assays

The Cell Counting Kit-8 (CCK-8) purchased from Dojindo Molecular Technologies, Inc., Kumamoto, Japan, was used to assess cell viability. LX-2 cells, which had been pre-treated with 10 ng/mL TGFβ-1 for 12 h, were seeded at a density of 2 × 10^4^ cells/well in the lower chamber of 24-well plates for the assay. UC-MSCs were seeded in the upper chamber at densities of either 2 × 10^4^ or 2 × 10^5^ cells/well and co-cultured with LX-2 cells for durations of 24–48 h. Additionally, for the MSC-CM treatment comparison, the LX-2 group, activated LX-2, and activated LX-2 with MSC-treatment were tested. Subsequently, 100 µL of CCK-8 solution was added to each well, and the cells were incubated for 1.5 h at 37 ℃. The optical absorbance of each well was measured at 450 nm utilizing the microplate reader. Wells containing culture medium without cells served as the blank controls.

### Peripheral blood mononuclear cell (PBMC) inhibition assay

PBMCs were adjusted to a concentration of 1 × 10^7^ cells/mL and labeled by incubating with 10 µM/mL carboxyfluorescein succinimidyl ester (CFSE) at 37℃ for 15 min. The labeled PBMCs were then plated at a density of 6 × 10^5^ cells per well. PBMCs primed with phytohemagglutinin (PHA) treatment served as the positive control. For the MSC-CM treatment, the primed PBMCs were incubated with MSC-CM for 5 days. Post-cultivation, PBMCs were collected and the lymphocyte proliferative capacities were evaluated using flow cytometry. The proportion of parental and daughter lymphocyte generations was calculated and analyzed using Modifit software. The total lymphocyte suppression rate was calculated using the formula: ([1 - sample daughter cell proportion] / daughter cell proportion of positive control) × 100%. All results were denoted as the mean ± standard deviation (SD).

### Statistical analysis

Descriptive and analytical statistics were conducted using SPSS software (ver. 25.0; USA, IL) and GraphPad Prism six. Data were presented as means ± SD with a minimum of three replicates for each measurement. One-way analysis of variance (ANOVA) for multiple comparisons and t-tests were used to assess significant differences. Values with a *p*-value less than 0.05 were considered statistically significant.

## Results

### Isolation, cultivation, proliferation, and characterization of UC-MSCs

**Fig. 1:**
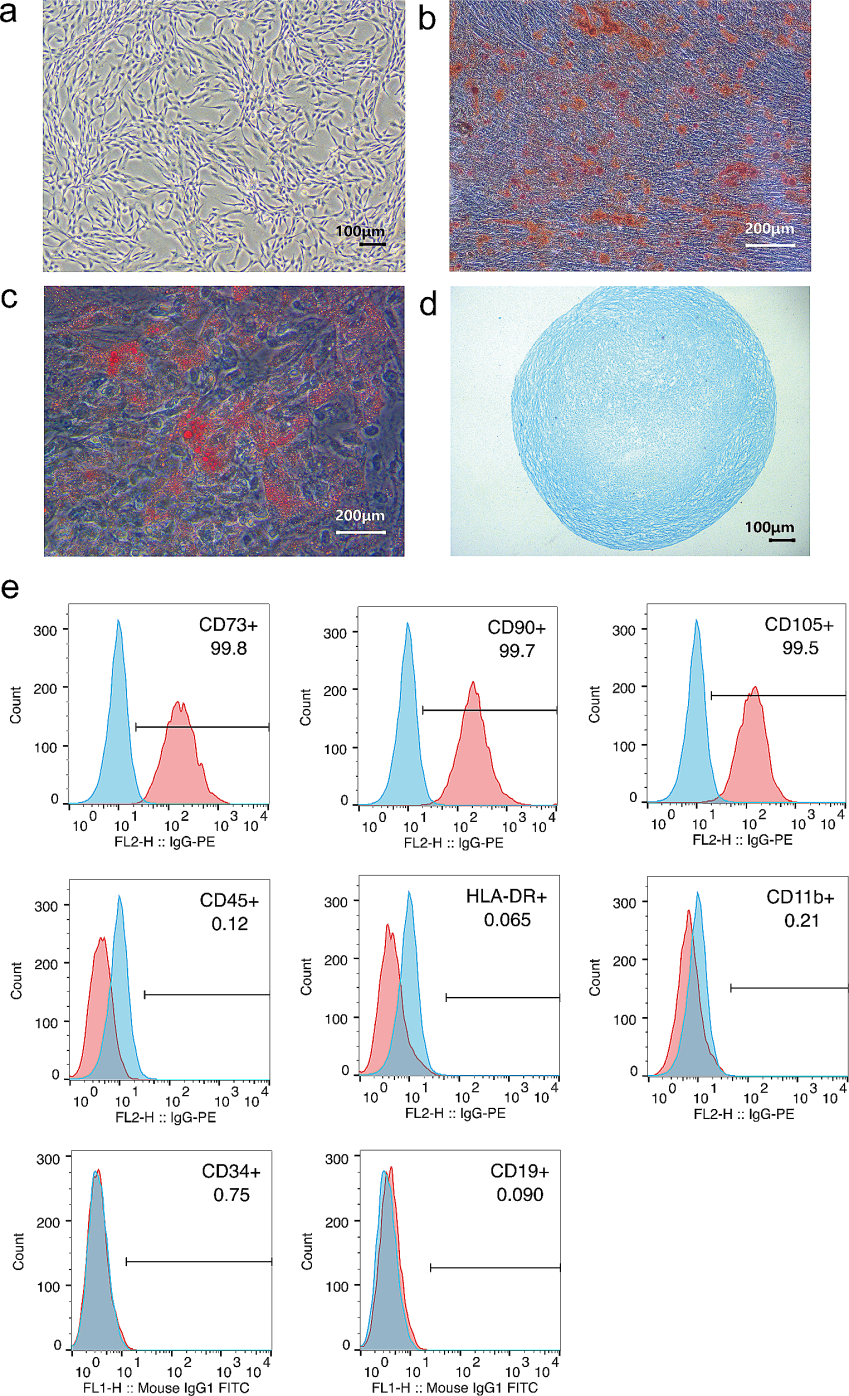
Phenotypic characteristics of UC-MSCs. (**a**) Light microscopic visualization of UC-MSCs (Passage 5), revealing their morphological features. (**b**) Alizarin red staining highlighting osteogenic differentiation of UC-MSCs. (**c**) Oil red-O staining emphasizing adipogenic differentiation of UC-MSCs. (**d**) Alcian blue stain depicting chondrogenic differentiation of UC-MSCs. (**e**) Flow cytometric analysis revealing the characteristic phenotype markers of MSCs

Initially, human UC-MSCs were isolated and meticulously characterized, exhibiting a distinct morphology with long spindle-shaped cells. These cells demonstrated proficiency in colony formation and efficient confluence (Fig. 1a). Furthermore, their multi-lineage differentiation potential was evident as they successfully differentiate into osteocytes, adipocytes and chondrocytes (Fig. 1b-d). Flow cytometry was utilized to analyze the phenotype of UC-MSCs, revealing a positive expression profile for markers CD73, CD90 and CD105. In contrast, the cells were negative for CD34, CD19, CD45, CD11b and HLA-DR (Fig. 1e). These observations strongly confirmed the successful generation and characterization of UC-MSCs. Furthermore, the cells strictly adhered to the criteria established by the International Society for Cellular Therapy for mesenchymal stem cells, encompassing both cellular purity and differentiation potential.

### Efficacy of MSC-CM and UC-MSCs in attenuating liver fibrosis in CCl_4_-induced rat models

**Fig. 2:**
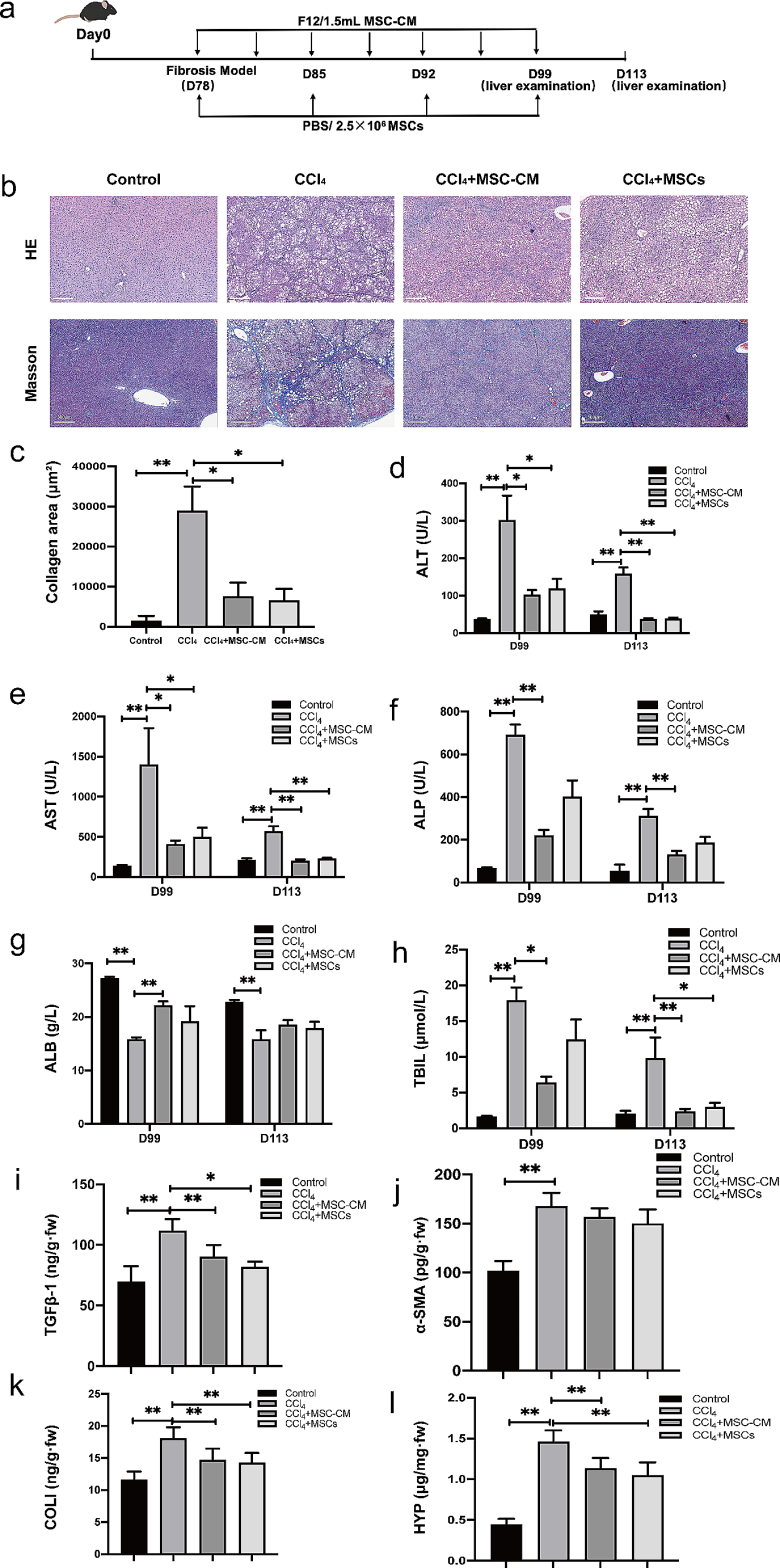
MSC-CM and UC-MSCs profoundly alleviate liver fibrosis in rat models. (**a**) Schematic representation of the administration timeline for intravenous injection of either MSC-CM or MSCs. Rats were administered bi-weekly CCl_4_ injections, in conjunction with specialized feed, until a successful fibrosis model was established. Following this, the animals received bi-weekly MSC-CM injections or weekly MSCs injections via the tail vein. Hepatic tissue samples and blood serum were collected on Day 99 and 113, corresponding to 3 weeks and 5 weeks post intravenous administration, respectively. (**b**) Representative images of HE staining and Masson’s trichrome staining are shown for hepatic sections obtained from oil-treated rats (Control), CCl_4_-induced fibrotic livers treated with PBS (CCl_4_), fibrotic rats treated with MSC-CM (CCl_4_ + MSC-CM), and fibrotic rats treated with UC-MSCs (CCl_4_ + MSCs). (**c**-**h**) Quantitative analysis of the fold changes in liver collagen content (**c**) and serum levels of ALT (**d**), AST (**e**), ALP (**f**), ALB (**g**) and TBIL (**h**) among different experimental groups is presented. (**i**-**l**) Alterations in the expression of hepatic fibrosis markers, including TGFβ-1, α-SMA, COL I, and HYP, were analyzed in each experimental group. Statistical significance was denoted as *P < 0.05, **P < 0.01, and ***P < 0.001

In our investigation to elucidate the therapeutic potential of MSC-CM and UC-MSCs against liver fibrosis, we utilized a liver fibrosis rat model induced by CCl_4_ administration combined with specialized feed. In this animal model, the combination of a high-salt, high-fat, and low-protein diet enhances the sensitivity of liver cells to CCl_4_, thereby inducing steatohepatitis with hepatocyte ballooning. Subsequently, the animals will progress gradually to liver fibrosis and ultimately to cirrhosis. This animal model enabled us to assess the remedial efficacy of both MSC-CM and UC-MSCs. The model group rats underwent bi-weekly subcutaneous injections of CCl_4_ oil solution for an eleven-week duration to induce fibrosis. Subsequently, rats in the MSC-CM treatment group were administered 1.5 mL of MSC-CM per rat bi-weekly, whereas those in the MSCs treatment group received weekly injections of 2.5 × 10^6^ cells per rat. Following the final dosage and two weeks after its administration, we evaluated liver pathology, fibrotic tissue areas, serum fibrosis markers and liver functionality, as depicted in Fig. 2a. Our findings revealed a significant reduction in liver fibrosis severity 5 weeks post-administration of both MSC-CM and MSCs. Histological analysis of liver sections using HE and Masson staining showed notably reduced fibrotic and collagen-rich regions in both the MSC-CM and MSCs group compared to control group (Fig. 2b-c). Elevated circulating concentrations of ALT, AST, ALP and TBIL, accompanied by diminished ALB levels, were characterized in the liver fibrosis rat model. However, both MSC-CM and MSCs administration effectively normalized the perturbations in ALT levels (Fig. 2d), AST (Fig. 2e) and TBIL levels (Fig. 2h). Specifically, MSC-CM treatment also ameliorated the perturbations in ALP and ALB levels, although no discernible differences were observed in the MSCs group (Fig. 2f-g). Subsequent serum analysis of HA, LN, PCIII and ColIV revealed that their levels were markedly elevated in fibrotic rats compared to the controls. However, treatments with both MSCs and MSC-CM could reverse these elevations, especially in LN levels (Table S1 and Table S2). We quantified assessed the protein concentration of various fibrosis-specific markers in liver tissues two weeks following the final administration. The findings revealed a marked elevation in the protein levels of TGFβ-1, α-SMA, COL1A, and HYP within the model group. Treatment with MSC-CM or MSCs led to a significant suppression of TGFβ-1, COL1A, and HYP protein expression in liver tissues (Fig. 2i-l and Table S3). However, no significant regulatory effect was observed for α-SMA expression.

In summation, these findings highlight the promising potential of both MSCs and MSC-CM injections in mitigating liver fibrosis progression in a CCl_4_-induced rat model. Notably, MSC-CM therapy exhibits similar anti-fibrotic efficacy to MSCs, indicating its potential as an effective treatment option.

### The MSC-CM mitigates activation of HSCs

**Fig. 3:**
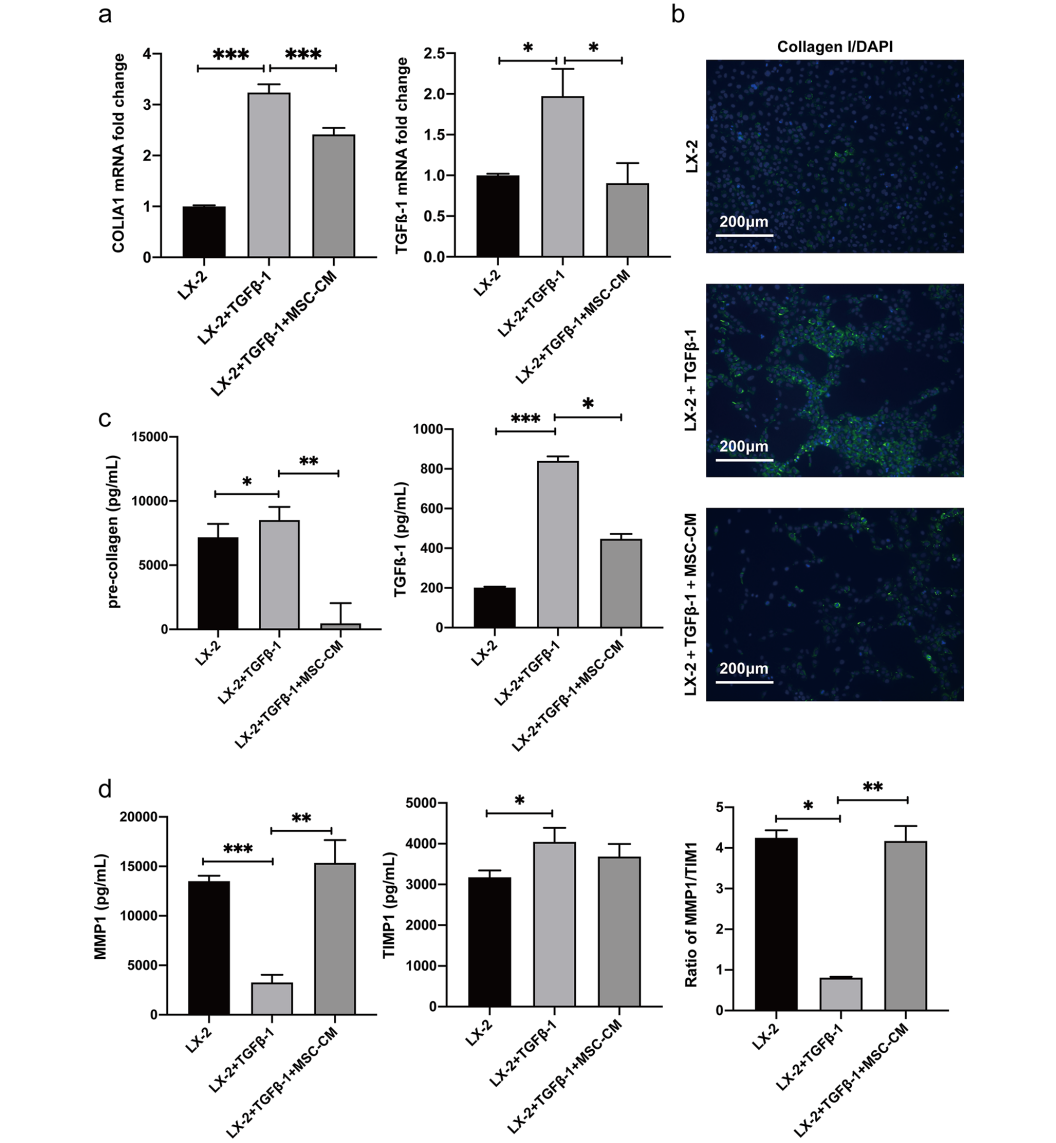
MSC-CM attenuates LX-2 activation. (**a**) qRT-PCR analysis of mRNA expression levels for COL1A1 and TGFβ-1 in LX-2 cultured alone (designated as LX-2 control), LX-2 activated with 10 ng/mL TGFβ1 (denoted as LX-2 + TGFβ-1) and treated with MSC-CM following TGFβ-1 activation (labled as LX-2 + TGFβ-1 + MSC-CM). (**b**) Immunofluorescence micrographs were captured to visualized the cells, with green fluorescence denoting collagen expression and blue fluorescence for nuclear staining. (**c**) The protein concentrations of pre-collagen and TGFβ-1 assessed by ELISA. (**d**) Quantification of MMP1 and TIMP1 protein measured by ELISA. All values were presented as mean ± SD, obtained from triplicate experiments. Statistical significance was as follows: *P < 0.05, **P < 0.01, and ***P < 0.001

Our findings in vivo highlighted the therapeutic potential of both MSC-CM and MSCs in ameliorating fibrosis in rats. To further elucidate the underlying mechanism of MSC-CM therapy, we employed TGFβ-1 to mimic the activation of HSCs in vitro. With TGFβ-1 treated for 12 h, there was a marked upregulation in the expression of COL1A1 and TGFβ-1, two crucial pro-fibrotic genes, compared to the control, both at mRNA and protein levels. Interestingly, when these activated LX-2 cells were exposed to MSC-CM for 48 h, there was a marked down regulation of COL1A1 and TGFβ-1, both transcriptionally (Fig. 3a) and translationally (Fig. 3b-c). Furthermore, our assay extended to the evaluation of mRNA and protein expressions of MMP1 and tissue inhibitor of metalloproteinases 1 (TIMP1). It was evident that MMP1 was considerably diminished in activated LX-2 cells, but this trend was effectively reversed by the intervention of MSC-CM (Fig. 3d). Additionally, the MSC-CM effectively restored the imbalance of the MMP1/TIMP1 ratio that was induced by TGFβ-1. Collectively, these findings underscore the consistent modulatory influence of MSC-CM on the activation status of LX-2 cells, suggesting its potential role in regulating fibrotic processes.

### UC-MSCs promote apoptosis and inhibit HSC activation

**Fig. 4:**
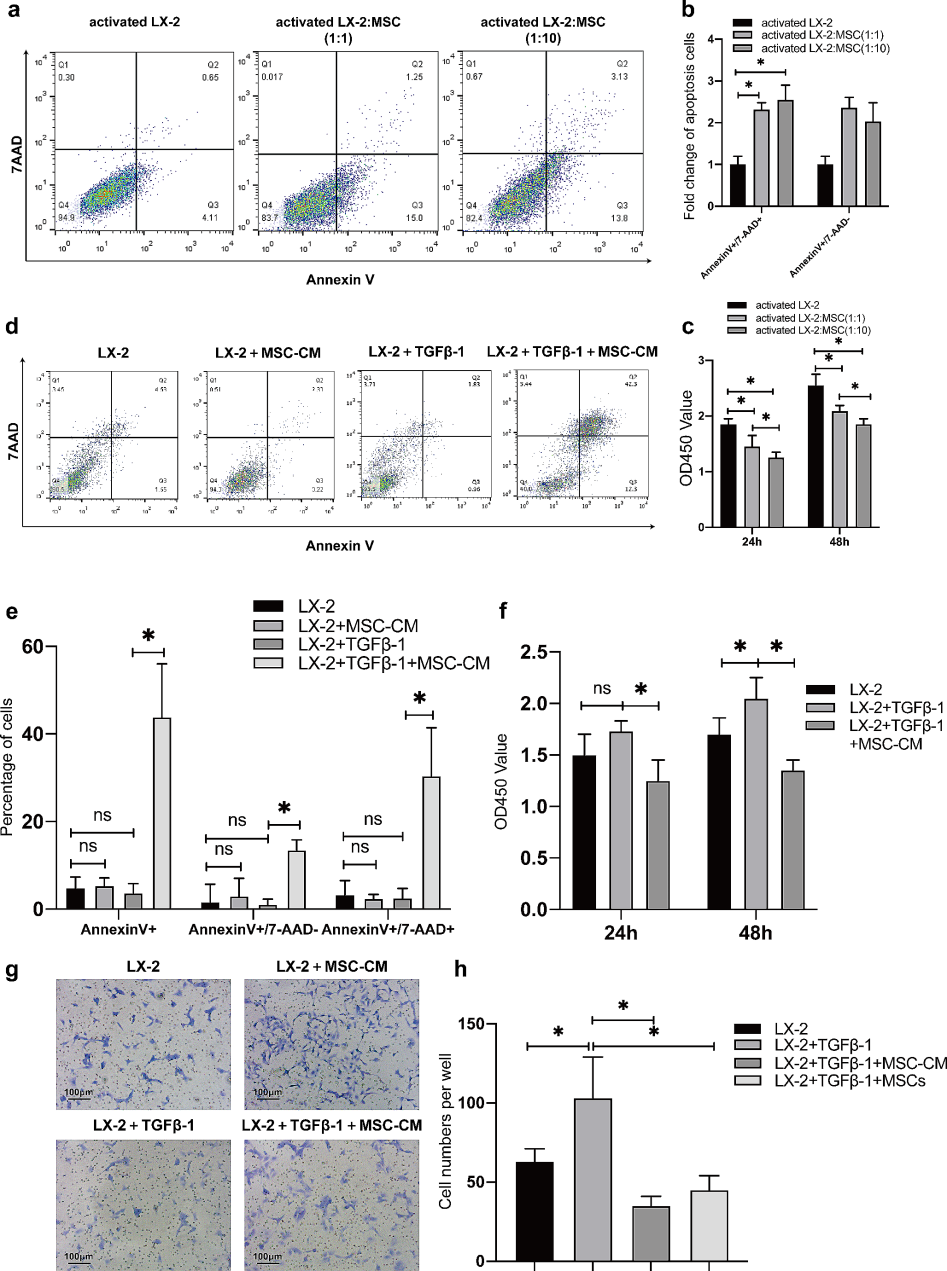
UC-MSCs modulate apoptosis and activation of activated HSCs. (**a**) Apoptosis in activated LX-2 cells with TGFβ-1 pre-treatment was significantly induced co-cultured with UC-MSCs for 48 h, as visualized by the fluorescent Annexin V/7-AAD assay. (**b**) Apoptosis induction was analyzed at varying target-effector ratios, with a 1:1 and 1:10 ratio of activated LX-2 cells to MSCs, revealing a proportional change in apoptotic cells. (**c**) Cell viability of activated LX-2 was evaluated using the CCK8 assay after co-culturing with varying concentrations of MSCs for 24 and 48 h. (**d**) A significant increase in apoptosis of activated LX-2 cells was observed 48 h post MSC-CM treatment, as demonstrated by fluorescent Annexin V/7-AAD assay. (**e**) A comparative analysis of apoptotic cell percentages was conducted among quiescent LX-2, quiescent LX-2 treated with MSC-CM, activated LX-2 and MSC-CM treated activated LX-2 cells. (f) Cell viability of LX-2, activated LX-2 with TGFβ-1 treatment and activated LX-2 with MSC-CM treatment was assessed over a period of 24 and 48 h. (**g**) A photograph depicting various HSCs groups, includingLX-2 alone, activated LX-2, activated LX-2 treated with MSC-CM, and MSC-cocultured activated LX-2). (**h**) Quantitative analysis of migrated cells across the distinct treatment groups. All experiments were performed in triplicate, and the data are presented as mean ± SD. *P < 0.05

To delineate whether UC-MSCs exert a direct influence on the proliferation and apoptosis of activated HSCs or whether their effects are mediated through secreted soluble factors in MSC-CM, LX-2 cells were initially activated with TGFβ-1 and subsequently co-cultured with UC-MSCs using transwells or directly treated with MSC-CM. We also prepared resting LX-2 without TGFβ-1 treatment and studied the impact of MSC-CM on their apoptosis. Following 48 h of co-culture at varying target-effector ratios (1:1 and 1:10), we observed a notable increase in apoptosis cells among the activated LX-2 (Fig. 4a-b). Specifically, there was approximately a three-fold increase in apoptosis rates, reaching 13.8 ± 2.2% at a 1:10 ratio and 15.0 ± 3.12% at a 1:1 ratio, when compared to the control (4.1 ± 0.26%). Moreover, when activated LX-2 were supplemented with MSC-CM for 48 h, a pronounced increase in apoptosis was observed, with 12.3 ± 2.3% in the MSC-CM-treated group versus 0.96 ± 0.2% in the activated LX-2 group. However, no significant differences in apoptosis rates were observed between quiescent and activated LX-2 cells. MSC-CM also didn’t influence the apoptosis of resting LX-2 cells that had not been previously exposed to TGFβ-1 treatment (Fig. 4d-e). Both co-culture with UC-MSCs (Fig. 4c) and MSC-CM treatment (Fig. 4f) led to a discernible reduction in HSC viability and proliferation, as assessed by the CCK8 assay. The effects of both UC-MSCs and MSC-CM on the migratory capabilities of activated LX-2 were evaluated. Both MSCs and MSC-CM reduced the number of cells migrating from the top to the bottom of the transwell chambers (Fig. 4g-h), which indicated that MSC could effectively inhibit the migration of activated LX2. Collectively, these findings emphasize that UC-MSCs can enhance activated HSCs apoptosis and attenuate HSC activation, achieved through both co-culture and MSC-CM treatment.

### MSC-CM exerts immunosuppressive effects on both HSCs and PBMCs

**Fig. 5:**
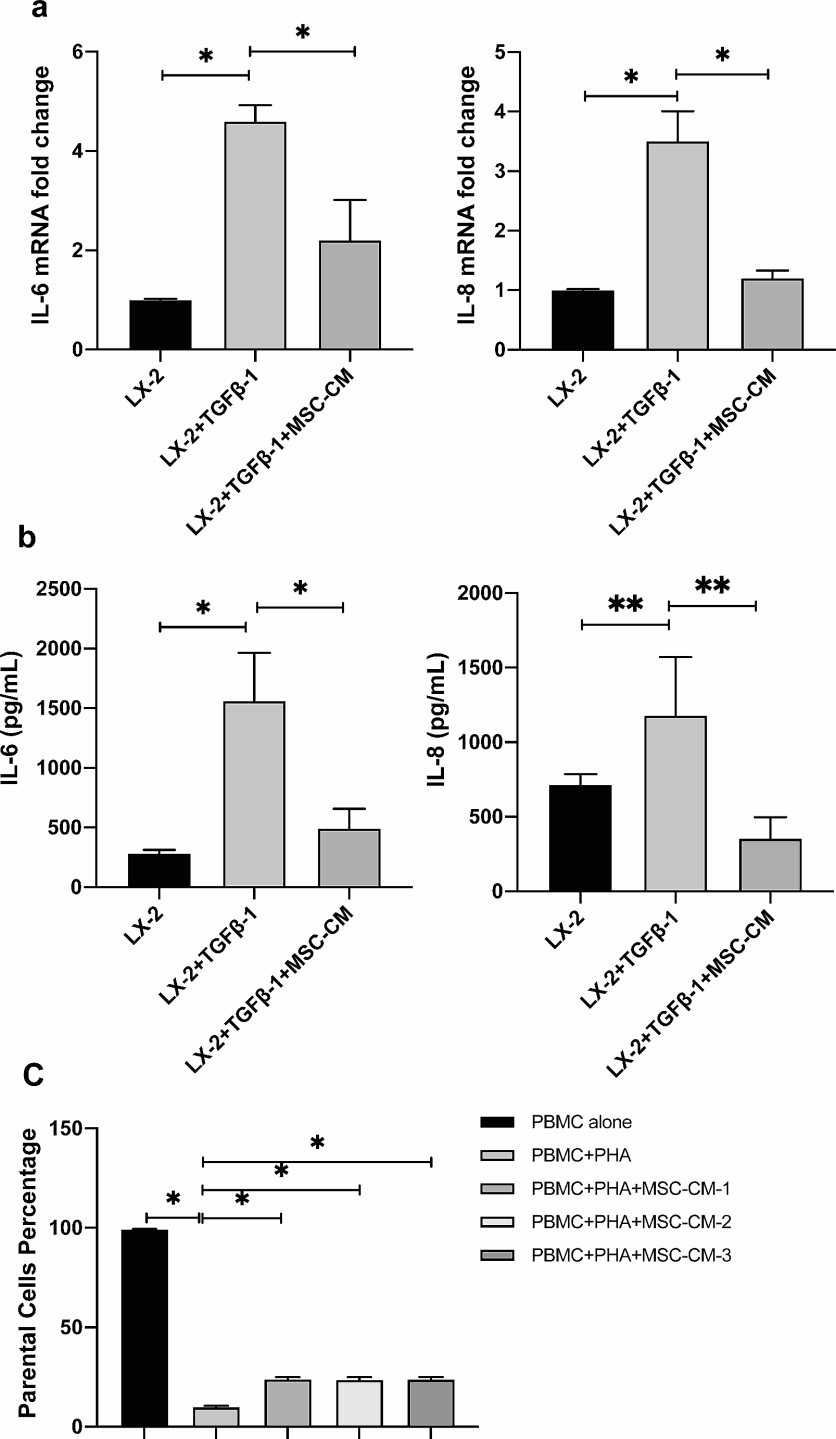
Immunosuppressive impact of MSC-CM on both HSCs and PBMCs. (**a**) qRT-PCR was utilized to assess the mRNA levels of IL-6 and IL-8 under three experimental conditions: untreated LX-2 cells serving as a control (designated as LX-2), TGFβ-1 stimulated LX-2 cells (designated as LX-2 + TGFβ-1), and MSC-CM treated activated LX-2 cells (designated as LX-2 + TGFβ-1 + MSC-CM). (**b**) The protein concentrations of IL-6 and IL-8 of different groups were quantified by ELISA. (**c**) CFSE staining was employed to determine the proportion of progenitor cells within native PBMCs, PBMCs activated with 10 µg/mL PHA, and activated PBMCs treated with three distinct MSC-CMs. Protein quantifications are presented as mean ± SD, with each experiment replicated thrice. ∗*P* < 0.05 and ∗∗*P* < 0.01

To elucidate the immunosuppressive capabilities of MSC-CM within the liver fibrosis, we examined the expression of two crucial inflammatory mediators, IL-6 and IL-8 in HSCs. We observed a significant upregulation of both mRNA and protein levels of IL-6 and IL-8 in activated LX-2 cells, as assessed by qRT-PCR (Fig. 5a) and ELISA assays (Fig. 5b). Intriguingly, MSC-CM administration effectively attenuated the secretion of IL-6 and IL-8 from these activated LX-2 cells, indicating a shift towards a less inflammatory phenotype. Concurrently, we performed an analysis to evaluate the inhibitory effects of MSC-CM on total lymphocyte proliferation. After the PHA stimulation, a significant decrease in the progenitor cell count of PBMCs was observed (*P* < 0.01), indicating successful lymphocyte activation. Notably, the suppressive effect on lymphocyte proliferation was more pronounced with three distinct MSC-CMs, ranging from 15.41 to 15.69% (*P* < 0.01) (Fig. 5c). Collectively, these observations emphasize the extensive immunomodulatory abilities of MSCs in liver fibrosis models. This immunomodulation occurs not merely through direct cellular interactions but also via bioactive soluble factors secreted by MSCs. The extent of this modulation extends beyond HSCs to encompass lymphocytes, further highlighting the diverse and widespread influence of MSCs in modulating immune responses.

## Discussion

Extensive evidence from clinical trials has validated the safety and therapeutic efficacy of MSC transplantation for treating liver diseases. Bone marrow-MSCs [16–18], AT-MSCs [19] and UC-MSCs [14, 20] have collectively demonstrated remarkable improvements in liver regeneration, liver function and patient quality of life. Nevertheless, it’s crucial to recognize that, upon introduction into the circulatory system, MSCs tend to localize within the lungs, presenting a potential risk of pulmonary embolism in specific preclinical research or clinical trials [15, 21]. Although MSCs primarily actions through immunomodulation and paracrine effects, living MSCs persistently release growth factors such as fibroblast growth factors (FGFs), transforming growth factors (TGFs), vascular endothelial growth factors (VEGFs) and epidermal growth factors (EGFs), which can potentially stimulate neo-vascularization [22]. Conversely, multiple studies have proposed that extracellular vesicles (EVs) derived from MSCs function as a key mechanism for tissue repair and inflammation mitigation [23, 24]. Also, more and more research have reported that hUC-MSCs inhibit fibrosis by up-regulating the expression of miR-148a-5p [25] or BMP7 through exosomes pathway [26]. However, the persisting uncertainty regarding exosome-specific biomarkers has resulted in a lack of standardized quality criteria for the development of therapeutic products. Given this, it is worthwhile to delve into the therapeutic efficacy of non-viable cell preparations. Specifically, MSC-CM might emerge as a compelling alternative to direct MSC therapy for various ailments. In our current exploration, we primarily focused on assessing the impact of MSC-CM administration in a rat model with CCL_4_-induced liver fibrosis. Firstly, we compared the cell number of MSCs that preparing MSC-CM and the number of UC-MSCs that directly injected to the mice. The remaining MSC cell counts were shown in Table S4. The live cell counts were resulting in an average value of 1.03 × 10^7^ cells. In animal experiments, the MSC-CM treatment group received bi-weekly injections of 1.5 mL of MSC-CM per rat. This corresponded to injecting the conditioned media equivalent to a maximum of 2 × 10^6^ MSC cells (calculated as 1.03E7 × 1.5 × 2 ÷ 15 = 2.06E6) per week. In contrast, the MSC-treatment group received 2.5E6 cells per rat once a week. Notably, the cell numbers used for MSC-CM preparation and MSC treatment were comparable in magnitude. Our findings indicated that both MSC-CM and MSCs can drive liver regeneration and alleviate the severity of fibrosis. However, when compared to MSCs treatment, MSC-CM exhibited a more pronounced suppressive effect on serum biochemical markers. On the other hand, only MSCs administration was able to significantly diminish LN expression levels among serological hepatic fibrosis markers. This discrepancy could potentially be attributed to variations in the frequency and duration of treatment during the investigation. By extending the administration dose and duration of MSC-CM administration, there may be a decrease in hepatic fibrosis markers. Notably, employing MSC-CM presents a simpler approach compared to direct MSCs administration.

Accumulating evidence confirms that MSCs exhibit tissue heterogeneity, originating from diverse sources such as bone marrow, adipose tissue, amniotic tissue, dental pulp, and umbilical cord. MSCs derived from these diverse sources exhibit unique characteristics in proliferation, differentiation, paracrine factor secretion, and immunomodulatory capabilities [27, 28]. In our study, we specifically chose UC-MSCs as the focus of our research and successfully isolated and cultivated them. UC-MSCs are favored due to the absence of significant ethical concerns, possess primitive characteristics, are easily accessible, and have a low risk of immunogenicity and viral transmission [20, 29]. HSCs play a pivotal role in linking hepatic inflammation to hepatic fibrosis in fibrotic liver diseases [30]. Many studies had demonstrated that hepatocyte growth factor (HGF) serves as a significant inhibitor of liver fibrosis. Additionally, MSCs were capable of secreting various cytokines, including HGF [31]. We examined the concentration of HGF in MSC-CM using Elisa assays. Notably, we observed abundant levels of HGF in the MSC-CMs (data not shown).

Multiple signaling pathways, encompassing TGF-β/Smad, PI3K/Akt, Notch, NF-κB, and Wnt/β-catenin, are implicated in the activation of HSCs and subsequent progression of liver fibrosis [32–35]. Our findings indicate that cytokines secreted in MSC-CM can inhibit TGFβ-1-induced HSC activation, as evidenced by the downregulation of pro-fibrotic markers such as COL1A1, TGFβ-1, and pre-collagen in LX-2 cells. Furthermore, in our observations, MSC-CM was found to elevate the expression of MMP1, thereby promoting enhanced ECM degradation and rectifying the MMP1/TIMP1 imbalance, ultimately supporting liver fibrosis resolution. Simultaneously, our study revealed that MSC-CM possesses immune regulatory functions and can suppress the secretion of inflammatory cytokines such as IL-6 and IL-8 during the fibrotic process in vitro. We hypothesize that MSCs primarily secrete HGF via paracrine signaling and exert therapeutic effects by mitigating liver fibrosis through inflammatory suppression.Several studies have hypothesized that MSCs can induce cell cycle arrest and apoptosis in activated HSCs, findings that our research validates [36]. Our investigations revealed that both co-culturing HSCs with MSCs and treating HSCs with MSC-CM effectively inhibited proliferation and enhanced apoptosis. Previous studies have shown that MSCs have the ability to regulate both the innate and adaptive immune response [2]. The liver, being a crucial immunological organ, hosts various immune cells including macrophages, Kupffer cells, dendritic cells, natural killer cells, T cells and B cells. In our investigation, we discovered that MSC-CM also exhibits suppressive effects on lymphocyte proliferation. MSCs and MSC-CM possess the potential to impact both LX-2 cells and immune cells. Given that the activation of hepatic stellate cells serves as the pivotal mechanism underlying liver fibrosis, LX-2 cells emerged as an ideal cellular model for examining liver fibrosis in vitro in this study. For the therapeutic potential of hUC-MSCs for acute-on-chronic liver injury (ACLI) and acute-on-chronic liver failure (ACLF) models, LO-2 or primary normal liver cells were more suitable [37]. Regarding immunomodulation, both MSCs and MSC-CM exhibit suppressive effects on the proliferation of activated PBMCs. The immunosuppressive capabilities of live MSCs surpass those of MSC-CM administration. This indicates that the soluble proteins secreted by MSCs possess an immunomodulatory function, thereby offering therapeutic potential for various autoimmune diseases.

## Conclusion

Our research ascertains the therapeutic efficacy of both MSC-CM and MSCs in fibrotic rat models. Furthermore, our data suggest that MSC-CM administration could potentially offer a more viable and effective alternative to MSC therapy for chronic liver fibrosis.

## Electronic supplementary material

Below is the link to the electronic supplementary material.

Supplementary Material 1

## Data Availability

No datasets were generated or analysed during the current study.
