# Supplementary material for: Human umbilical cord-derived mesenchymal stem cells attenuate hepatic stellate cells activation and liver fibrosis
Source: Mol Biol Rep. 2024 Jun 14;51(1):734. doi: 10.1007/s11033-024-09664-6 (PMC11178641; doi:10.1007/s11033-024-09664-6)
Supplement: Supplementary file 1 — Supplementary Material 1 [file 11033_2024_9664_MOESM1_ESM.docx]

***Supplementary materials***

**Human Umbilical Cord-Derived Mesenchymal Stem Cells Attenuate Hepatic Stellate Cells Activation and Liver Fibrosis**

**Table S1.** Quantitative analysis of serological markers indicative of hepatic fibrosis on 3 weeks.

| On 3 weeks | N | COLⅣ  (ng/mL) | PCⅢ  (ng/mL) | LN  (ng/mL) | HA  (ng/mL) |
| --- | --- | --- | --- | --- | --- |
| Control | 10 | 5.01±0.22 | 6.81±0.42 | 60.94±2.03 | 77.67±2.40 |
| CCL4 Model | 8 | 8.11±0.34^△△^ | 20.83±0.61^△△^ | 107.70±4.08^△△^ | 122.56±6.01^△△^ |
| MSC-CM | 10 | 7.15±0.25 | 19.70±0.62 | 98.77±3.47 | 119.84±4.00 |
| MSCs (2.5×10^6^) | 10 | 6.99±0.48 | 20.24±0.57 | 99.06±3.18 | 117.05±5.11 |

**Table S2.** Quantitative analysis of serological markers indicative of hepatic fibrosis on 5 weeks.

| **On 5 weeks** | **N** | **COLⅣ**  **(ng/mL)** | **PCⅢ**  **(ng/mL)** | **LN**  **(ng/mL)** | **HA**  **(ng/mL)** |
| --- | --- | --- | --- | --- | --- |
| Control | 10 | 4.30±0.14 | 5.24±0.23 | 46.48±2.88 | 68.53±2.74 |
| CCL4 Model | 8 | 7.22±0.28^△△^ | 18.22±0.64^△△^ | 99.70±4.06^△△^ | 97.61±7.47^△△^ |
| MSC-CM | 10 | 6.19±0.47 | 18.07±0.71 | 89.45±3.86 | 93.28±5.01 |
| MSCs (2.5×10^6^) | 10 | 6.54±0.29 | 17.09±0.55 | 84.70±3.43^**^ | 93.42±2.25 |

Note: Compared to control, ^ΔΔ^*P*≤0.01; compared to CCL4 model, ^*^*P*≤0.05, ^**^*P*≤0.01.

**Table S3.** Quantitative analysis of protein of hepatic fibrosis tissue on 5 weeks.

| On 5 weeks | N | TGF-β1 | α-SMA | COLI | HYP |
| --- | --- | --- | --- | --- | --- |
|  |  | （ng/g.fw） | （pg/g.fw） | （ng/g.fw） | （μg/mg.fw） |
| Control | 10 | 69.54±13.00 | 101.95±9.94 | 11.61±1.28 | 0.45±0.07 |
| CCL4 Model | 8 | 111.52±9.80^ΔΔ^ | 167.78±13.65^ΔΔ^ | 18.03±1.77^ΔΔ^ | 1.46±0.14^ΔΔ^ |
| MSC-CM | 10 | 90.32±9.6^**^ | 156.91±8.62 | 14.66±1.80^**^ | 1.13±0.13^**^ |
| MSCs (2.5×10^6^) | 10 | 82.00±4.25^*^ | 150.12±14.35 | 14.27±1.51^*^ | 1.05±0.16^**^ |

Note: Compared to control, ^ΔΔ^*P*≤0.01; compared to CCL4 model, ^*^*P*≤0.05, ^**^*P*≤0.01.

**Table S4.** Live cell counts of MSCs remained in the flasks after MSC-CM collection.

|  | Sample 1 | Sample 2 | Sample 3 | Sample 4 | Mean |
| --- | --- | --- | --- | --- | --- |
| Cell Count | 9.8E6 | 1.13E7 | 9.7E6 | 1.04E7 | 1.03E7 |
